# Predicting the occurrence of venous thromboembolism: construction and verification of risk warning model

**DOI:** 10.1186/s12872-020-01519-9

**Published:** 2020-05-27

**Authors:** Chen Shen, Binqian Ge, Xiaoqin Liu, Hao Chen, Yi Qin, Hongwu Shen

**Affiliations:** 1 Department of Nursing, Affiliated Hospital of Nantong University, 20 Xisi Road, Nantong City, 226000 Jiangsu China; 2 School of Nursing, Suzhou Vocational Health College, 28 Kehua Road, Suzhou City, 215009 Jiangsu China; 3 Department of Information, Affiliated Hospital of Nantong University, 20 Xisi Road, Nantong City, 226000 Jiangsu China

**Keywords:** Venous thromboembolism, Risk factors, Caprini scale, Logistic regression analysis, Predictive model

## Abstract

**Background:**

The onset of venous thromboembolism is insidious and the prognosis is poor. In this study, we aimed to construct a VTE risk warning model and testified its clinical application value.

**Methods:**

Preliminary construction of the VTE risk warning model was carried out according to the independent risk warning indicators of VTE screened by Logistic regression analysis. The truncated value of screening VTE was obtained and the model was evaluated. ROC curve analysis was used to compare the test of Caprini risk assessment scale and VTE risk warning model. The cut-off value of the VTE risk warning model was used to evaluate the test effectiveness of the model for VTE patients with validation data set.

**Results:**

The VTE risk warning model is p = e^x^ / (1+ e^x^), x = − 4.840 + 2.557 • X_10(1)_ + 1.432 • X_14(1)_ + 2.977 • X_15(1)_ + 3.445 • X_18(1)_ + 1.086 • X_25(1)_ + 0.249 • X_34_ + 0.282 • X_41_. ROC curve results show that: AUC (95%CI), cutoff value, sensitivity, specificity, accuracy, Youden index, Caprini risk assessment scale is 0.596 (0.552, 0.638), 5, 26.07, 96.50, 61.3%, 0.226, VTE risk warning model is 0.960 (0.940, 0.976), 0.438, 92.61, 91.83, 92.2%, 0.844, respectively, with statistically significant differences (Z = 14.521, *P* < 0.0001). The accuracy and Youden index of VTE screening using VTE risk warning model were 81.8 and 62.5%, respectively.

**Conclusions:**

VTE risk warning model had high accuracy in predicting VTE occurrence in hospitalized patients. Its test performance was better than Caprini risk assessment scale. It also had high test performance in external population.

## Background

Venous thromboembolism (VTE) is a common disease with high morbidity and mortality [1], including deep vein thrombosis (DVT) and pulmonary embolism (PE), VTE is the third most common cardiovascular disorder. The incidence of VTE is concealed and its prognosis is poor. At the same time, the increasing incidence rate showed a trend of younger patients [2, 3]. We should focus not only on the many influencing factors of VTE [4–8], but also on its early identification and early intervention [9, 10]. Studies have shown a lack of clinical VTE care standards for inpatients and the low VTE prevention rate which indicate that further improvement is needed [11]. Approximately 50% of VTEs are provoked by immobilization, trauma, surgery, or hospitalization in previous 3 months [12–15], and 20% associated with cancer while 30% unprovoked [16–18]. VTE has many risk factors which are constantly multiplied [19–25]. Currently, Caprini Risk Assessment Scale is widely used in clinical practice. However, genetic and environmental differences between Western and China limit the validity of Caprini Risk Assessment Scale in Chinese patients. Medical records which contain rich information about disease progression, are useful in mining new risk factors related to VTE patients. Each patient will undergo a series of laboratory tests upon admission, and the blood test indicators of VTE patients will be abnormal in varying degrees [26–28]. The timely detection of abnormal change will facilitate the VTE occurrence risk assessment and enable early warning and intervention. Therefore, based on characteristics of VTE patients in China, this study screened out VTE risk early warning indicators other than the traditional scale, and established a VTE Risk Warning Model. This study was to effectively achieve the primary prevention of VTE and provide a scientific theory for VTE prevention.

## Methods

### Study population

The study conducted from January 1, 2017 to June 30, 2018 in Affiliated Hospital of Nantong University. The inclusion criteria were as follows: patients diagnosed with VTE during hospitalization, age ≥ 18 years, hospitalization time ≥ 2 days (48 h), and clinical medical records completed. Patients who had VTE before admission, those with superficial vein thrombosis, and those who used anticoagulants were excluded. Finally, we included 257 VTE patients. Two hundred fifty-seven age- and disease-duration- matched non-VTE patients of the same period were also included in this study and built the modeling data set. In addition, 63 VTE patients and 85 non-VTE patients from July 1, 2018 to December 31, 2018, were selected for a validation data set, identical inclusion criteria with the former modeling data set. This clinical research protocol complies with relevant provisions of Helsinki Declaration on the protection of the rights and interests of subjects.

### Study design

The modeling data set was analyzed by T / *χ*^2^ test and Logistic regression analysis. According to VTE independent risk factors screened by Logistic regression analysis, a VTE risk warning model was constructed, and the cut-off value of VTE screening obtained. The cut-off value of VTE risk warning model was used to verify the screening efficacy of VTE risk warning model for VTE patients in the validation data set.

### Assessments

Since Logistic regression using maximum likelihood estimation (MLE) method for regression coefficient estimation is sensitive to multicollinearity, a high degree of multicollinearity will lead to a great change in coefficient estimation value or symbol. Therefore, multicollinearity analysis needed to be carried out before multivariate analysis. The variance expansion coefficient (VIF) diagnostic method is one of the common methods. Generally, VIF > 5 indicates multicollinearity existence. In a large sample, VIF > 10.

The Gold Standard is required in Diagnose Test to distinguish the experimental group and control group. The effectiveness of VTE risk early warning model was evaluated by four-division table of diagnostic data. The evaluation indicators mainly include Sensitivity (Sen), Specificity (Spe), Accuracy (Acc), and Youden Index (Youden’ s Index). Receiver operating characteristic curve (ROC) is a widely accepted criterion. The area under curve (AUC) below 0.6 means low discrimination, 0.6 to 0.75 medium discrimination, and above 0.75 high discrimination. The high AUC represents high model accuracy.

### Statistical analysis

Statistical analysis adopted SPSS 20.0. In univariate analysis, all statistical variables of VTE group were compared with corresponding variables of control group in order to determine the *P* value of all statistical variables. Measurement data were described by (^−^*X ± S*), and count data by frequency. The categorical variables were tested by *χ*^2^ test. The calibration was performed by*χ*^2^ test or Fisher exact probability method. And continuous variables were tested by *t* test or *t*^2^ test. Logistic regression was used in multivariate analysis. In this model, the variables were selected based on the results of univariate analysis. Variables in univariate analysis that were hypothesis-tested *P* < 0.3 (in order to prevent missing possible early warning indicators) and consistent with previous documents and clinical experience were included in the follow-up multivariate analysis. In order to simplify the model, stepwise regression method was adopted to screen model variables. The regression method was set as “Forward: LR”, and test level *α* = 0.05 was specified for introducing variables into the model and 0.10 for removing variables from the model. Logistic regression was used to obtain the regression coefficient, standard error, chi-square value of Wald, *P* value, corresponding OR value and 95% confidence interval of the possible predictors. The independent risk warning index of VTE screened by Logistic regression analysis was used to construct the VTE risk warning model, and its screening efficiency was evaluated and compared by ROC. External validation of VTE risk warning model was carried out by using the four-division of diagnostic data to evaluate its test effectiveness. *P* < 0.05 was considered statistically significant.

## Results

### Univariate analysis of VTE risk warning indicators

According to relevant literature and clinical practice, the VTE risk warning indicators which are not included in Caprini score scale mainly include four parts as following: (1) General indicators, including gender, patient origin, nationality, payment methods, length of stay (days); (2) Related indicators of current medical history, including 17 variables such as cough, expectoration, hemoptysis, dyspnea, pleural chest pain, cyanosis, pain in the precardiac area, palpitations, shortness of breath after exertion, chest tightness and shortness of breath, syncope with unknown cause, pleural effusion, unilateral lower limb Pain, deep venous tenderness in the lower limbs, pigmentation in the lower limbs, walking fatigue in the lower limbs, and increased local skin temperature in the lower limbs; (3) Relevant indicators of previous history, mainly including 7 variables such as hypertension, diabetes, smoking, systemic connective tissue disease, renal insufficiency, liver disease (hepatitis or liver damage), anemia; (4) The relevant indexes of the laboratory inspection items, mainly including 11 variables such as prothrombin time (PT), thrombin time (TT), activated partial thrombin time (APTT), Fibrinogen (FIB), Fibrinogen Degradation Product (FDP), International Normalized Ratio (INR), D-Dimerization, Albumin, platelet count, white blood cell count, number of red blood cells. In addition, we took the Caprini score as a risk warning indicator in the univariate analysis. The detailed results are shown in Table 1.

**Table 1:** Single factor analysis of VTE risk warning indicators in modeling dataset

Variables	No.		VTE group	Control group	*χ*^*2*^or *t*	*P*
			n(%)or^−^*X ± S*	n(%)or^−^*X ± S*		
Gender	X_1_	Male	109 (42.4)	123 (47.9)	1.540	0.215
		Female	148 (57.6)	134 (52.1)		
Patient source	X_2_	City	142 (55.3)	162 (63.0)	3.221	0.073
		Countryside	115 (44.7)	95 (37.0)		
Native place	X_3_	Native	249 (96.9)	253 (98.4)	1.365	0.243
		Non-native	8 (3.1)	4 (1.6)		
Payment method	X_4_	Health Insurance	111 (43.2)	138 (53.7)	5.679	0.017^*^
		Self-paying	146 (56.8)	119 (46.3)		
Length of stay (days)	X_5_		13.28 ± 6.64	13.00 ± 19.96	−2.998	0.003^*^
Cough	X_6_	No	218 (84.8)	197 (76.7)	5.517	0.019^*^
		Yes	39 (15.2)	60 (23.3)		
Expectorant	X_7_	No	227 (88.3)	246 (95.7)	9.568	0.002^*^
		Yes	30 (11.7)	11 (4.3)		
Hemoptysis	X_8_	No	244 (94.9)	251 (97.7)	2.678	0.102
		Yes	13 (5.1)	6 (2.3)		
Difficulty breathing	X_9_	No	250 (97.3)	253 (98.4)	0.836	0.361
		Yes	7 (2.7)	4 (1.6)		
Pleural chest pain	X_10_	No	230 (89.5)	251 (97.7)	14.28	<0.001^*^
		Yes	27 (10.5)	6 (2.3)		
Cyanosis	X_11_	No	244 (94.9)	254 (98.8)	6.451	0.011^*^
		Yes	13 (5.1)	3 (1.2)		
Anterior cardiac pain	X_12_	No	250 (97.3)	255 (99.2)	1.809	0.176
		Yes	7 (2.7)	2 (0.8)		
Palpitations	X_13_	No	251 (97.7)	252 (98.1)	0.093	0.761
		Yes	6 (2.3)	5 (1.9)		
Shortness of breath after exertion	X_14_	No	245 (95.3)	252 (98.1)	2.981	0.084
		Yes	12 (4.7)	5 (1.9)		
Chest tightness and shortness of breath	X_15_	No	184 (71.6)	244 (94.9)	50.272	<0.001^*^
		Yes	73 (28.4)	13 (5.1)		
Unexplained syncope	X_16_	No	238 (92.6)	254 (98.8)	12.157	<0.001^*^
		Yes	19 (7.4)	3 (1.2)		
Pleural effusion	X_17_	No	241 (93.8)	247 (96.1)	1.458	0.227
		Yes	16 (6.2)	10 (3.9)		
Unilateral lower limb pain	X_18_	No	201 (78.2)	255 (99.2)	56.671	<0.001^*^
		Yes	56 (21.8)	2 (0.8)		
Deep vein tenderness in lower limbs	X_19_	No	253 (98.4)	256 (99.6)	0.808	0.369
		Yes	4 (1.6)	1 (0.4)		
Lower extremity pigmentation	X_20_	No	255 (99.2)	257 (100)	0.502	0.479
		Yes	2 (0.8)	0 (0)		
Lower limb walking fatigue	X_21_	No	256 (99.6)	254 (98.8)	0.252	0.616
		Yes	1 (0.4)	3 (1.2)		
Local lower skin temperature increase	X_22_	No	252 (98.1)	256 (99.6)	1.518	0.218
		Yes	5 (1.9)	1 (0.4)		
Hypertension	X_23_	No	169 (65.8)	197 (76.7)	7.439	0.006^*^
		Yes	88 (34.2)	60 (23.3)		
Diabetes	X_24_	No	231 (89.9)	240 (93.4)	2.056	0.152
		Yes	26 (10.1)	17 (6.6)		
Smoking	X_25_	No	200 (77.8)	217 (84.4)	3.672	0.055
		Yes	57 (22.2)	40 (15.6)		
Systemic connective tissue disease	X_26_	No	255 (99.2)	252 (98.1)	0.579	0.447
		Yes	2 (0.8)	5 (1.9)		
Renal insufficiency	X_27_	No	248 (96.5)	254 (98.8)	3.072	0.08
		Yes	9 (3.5)	3 (1.2)		
Liver disease	X_28_	No	234 (91.1)	220 (85.6)	2.641	0.104
		Yes	23 (8.9)	37 (14.4)		
anemia	X_29_	No	246 (95.7)	250 (97.3)	0.921	0.337
		Yes	11 (4.3)	7 (2.7)		
PT (s)	X_30_		12.95 ± 7.29	12.66 ± 7.73	0.438	0.662
TT(s)	X_31_		18.28 ± 3.75	17.72 ± 1.58	2.206	0.028^*^
APTT(s)	X_32_		29.47 ± 6.76	30.20 ± 5.94	−1.300	0.194
FIB(g/L)	X_33_		2.84 ± 0.84	2.83 ± 0.89	0.131	0.896
FDP	X_34_		23.16 ± 28.85	5.28 ± 5.78	9.742	<0.001^*^
INR	X_35_		1.15 ± 1.23	1.09 ± 0.81	0.653	0.514
D-Dimer (mg/L)	X_36_		8.55 ± 14.37	1.37 ± 2.14	7.923	<0.001^*^
albumin (g/L)	X_37_		36.88 ± 5.05	37.01 ± 5.10	−0.290	0.772
Platelet (*10^9^/L)	X_38_		193.57 ± 74.38	188.94 ± 81.93	0.671	0.503
WBC(*10^9^/L)	X_39_		7.44 ± 2.81	6.42 ± 3.27	3.793	<0.001^*^
RBC(*10^9^/L)	X_40_		4.43 ± 3.66	4.23 ± 0.64	0.863	0.389
Caprini score	X_41_		4.60 ± 2.72	3.56 ± 1.13	5.661	<0.001^*^

*Note: *Statistically significant at 0.05 leve

### Multivariate analysis of VTE risk early warning indicators

Univariate analysis was performed on 41 variables, of which there were 15 variables with statistical significance of *P* < 0.05. In order not to omit possible VTE risk early warning related variables, increase the sensitivity of risk early warning model and allow more possible variables to be included in the variable, the variables with *P* < 0.3 in the univariate analysis or consistent with literature reports and clinical experience were included in the subsequent multivariate analysis. Therefore, we adopted a total of 28 variables. After colinear analysis, all variables had VIF less than 3, it can be considered no co-linearity among VTE risk warning indicators, which can be included in multi-factor logistic regression analysis, as shown in Table 2.

**Table 2:** Colinearity analysis of 28 VTE risk warning indicators including multivariate analysis

Variables		VIF	Variables		VIF
Gender	X_1_	1.406	Pleural effusion	X_17_	1.134
Patient source	X_2_	2.266	Unilateral lower limb pain	X_18_	1.205
Native place	X_3_	1.096	Local lower skin temperature increase	X_22_	1.062
Payment method	X_4_	1.123	Hypertension	X_23_	1.146
Length of stay (days)	X_5_	2.343	Diabetes	X_24_	1.113
Cough	X_6_	2.322	Smoking	X_25_	1.402
Expectorant	X_7_	2.302	Renal insufficiency	X_27_	1.054
Hemoptysis	X_8_	1.056	Liver disease	X_28_	1.065
Pleural chest pain	X_10_	1.122	TT(s)	X_31_	1.132
Cyanosis	X_11_	1.061	APTT(s)	X_32_	1.081
Anterior cardiac pain	X_12_	1.045	FDP	X_34_	1.767
Shortness of breath after exertion	X_14_	1.040	D-Dimer (mg/L)	X_36_	1.765
Chest tightness and shortness of breath	X_15_	1.148	WBC(*10^9^/L)	X_39_	1.138
Unexplained syncope	X_16_	1.106	Caprini score	X_41_	1.143

The above 28 variables with *P* < 0.3 were included in Logistic regression analysis, and up to 7 independent risk warning indicators were screened out, namely pleural chest pain X_10_ (*P* < 0.001), shortness of breath after exercise X_14_ (*P* = 0.045), Chest tightness and shortness of breath X_15_ (*P* < 0.001), unilateral lower extremity pain X_18_ (*P* < 0.001), smoking X_25_ (*P* = 0.005), fibrinogen degradation product X_34_ (*P* < 0.001), Caprini score X_41_ (*P* = 0.004). The logistic regression was used to obtain regression coefficient, standard error, Wald chi-square value, *P* value, its corresponding OR value, and 95% confidence interval of the independent risk warning indicators, as shown in Table 3.

**Table 3:** Logistic regression parameter estimation of patients in VTE group and control group

Variables	Point estimation	Standard error	*Wald chi-square values*	*P*	OR value point estimation	Lower interval	Upper interval
*X*_10_	2.557	0.624	16.800	<0.001	12.893	3.797	43.784
*X*_14_	1.432	0.713	4.029	0.045	4.185	1.034	16.935
*X*_15_	2.977	0.420	50.344	<0.001	19.622	8.623	44.653
*X*_18_	3.445	0.882	15.274	<0.001	31.352	5.571	176.454
*X*_25_	1.086	0.384	8.023	0.005	2.963	1.397	6.284
*X*_34_	0.249	0.024	106.673	<0.001	1.282	1.223	1.344
*X*_41_	0.282	0.099	8.184	0.004	1.326	1.093	1.608

### Construction of VTE risk warning model

According to above results The model independent variable assignment method was shown in Table 4. The final VTE risk warning model was as follows:

$$ \mathrm{p}={\mathrm{e}}^{\mathrm{x}}/\left(\ 1+{\mathrm{e}}^{\mathrm{x}}\ \right), $$

$$ \mathrm{x}=-4.840+2.557\bullet {\mathrm{X}}_{10(1)}+1.432\bullet {\mathrm{X}}_{14(1)}+2.977\bullet {\mathrm{X}}_{15(1)}+3.445\bullet {\mathrm{X}}_{18(1)}+1.086\bullet {\mathrm{X}}_{25(1)}+0.249\bullet {\mathrm{X}}_{34}+0.282\bullet {\mathrm{X}}_{41} $$

**Table 4:** The way to evaluate the value of the clinical variable of the VTE risk warning model

Clinical variables	No.	Assignment
Pleural chest pain	X_10_	No = 0, 1 = Yes
shortness of breath after fatigue	X_14_	No =0, 1 = Yes
chest dull shortness of breath	X_15_	No =0, 1 = Yes
unilateral lower limb pain	X_18_	No =0, 1 = Yes
smoking	X_25_	No =0, 1 = Yes
FDP	X_34_	Continuity variable
Caprini score	X_41_	Continuity variable
Outcome variables	Y	The control group =0, 1 = VTE group

Where e was the logarithm of natural numbers;

Pleural chest pain X_10_, shortness of breath after exercise X_14_, chest tightness and shortness of breath X_15_, unilateral lower extremity pain X_18_, smoking X_25_ and other variables were binary values (not specific medical history, 1 for yes, 0 for none). The unit of fibrinogen degradation product (X_34_) was (μg/ml). Caprini score (X_41_) was based on Caprini risk assessment scale, with no unit.

### Evaluation and comparison of VTE risk warning model test efficacy

According to VTE risk warning model formula, the predicted probability of VTE occurrence was calculated by ROC curve analysis. The area under ROC curve (AUC) was 0.960 (95% CI: 0.940, 0.976), the standard error was 0.009, and Z = 52.279. The Hosmer-Lemeshow test (H-L test) was performed on the VTE risk warning model, and the *χ2* was 55.441.

**Fig. 1:**
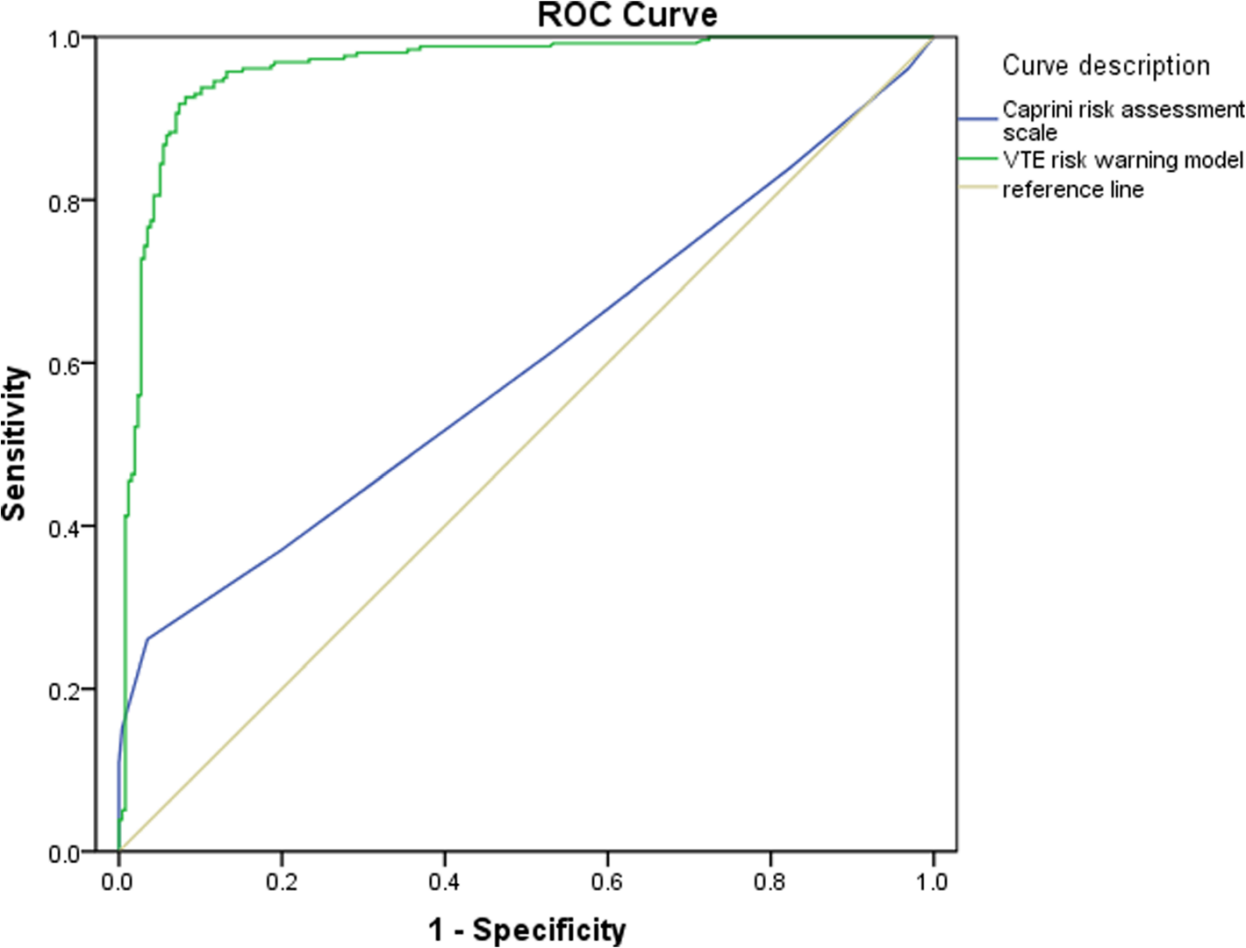
Comparison of ROC curves of VTE screening by Caprini risk assessment scale and VTE risk warning model

Caprini risk assessment scale and VTE risk warning model were used to predict the VTE truncation value (95% CI), which were 5 (4,5), 0.438 (0.263, 0.504), respectively. The VTE sensitivity was predicted to be 26.1 and 92.6% each, specificity 96.5 and 91.8%, accuracy 61.3 and 92.2%, and Youden index 0.23 and 0.84. AUC values were 0.596 (95%CI: 0.552, 0.638) and 0.960 (95%CI: 0.940, 0.976). The difference between above two groups was statistically significant (Z = 14.521, *P* < 0.0001), as shown in Fig. 1.

### External validation of VTE risk warning model

The validation data set included 63 VTE patients and 85 non-VTE patients. There was no significant difference in the distribution of general clinical variables between validation data set and modeling data set (*P* > 0.05), which avoided the deviation of results due to uneven distribution of clinical variables.

The validation data set was substituted into the established VTE risk warning model formula to calculate the prediction probability of the occurrence of VTE in each patient, and the model truncation value was used to evaluate the efficiency prediction of validated data set. The sensitivity was 77.8%, specificity 84.7%, accuracy 81.8% and Youden index 0.625. It indicated that VTE risk warning model had a higher prediction efficiency both in the internal population and external population.

## Discussion

### VTE risk early warning model

In this study, multivariate logistic regression analysis was performed on general information, medical history data, blood parameters, and Caprini scores of 257 VTE patients to screen out VTE risk warning indicators other than Caprini assessment scale. A clinical diagnostic model was developed, including 4 medical history data, 2 laboratory data, and 1 scale score. The area under ROC curve (AUC) of this model was 0.960 (95%CI: 0.940, 0.976). A good disease risk prediction model was not just a simple mathematical combination of dependent and independent variables, but also had actual clinical importance behind it. Our original intention was to reach high prediction efficiency, differentiation ability and sensitivity of the prediction model. The most commonindicator evaluating the discriminability of prediction models was AUC, also known as the C statistic. The larger AUC, the better the discriminant ability of the prediction model. AUC < 0.6 indicates poor differentiation, 0.6–0.75 certain differentiation ability, and > 0.75 good differentiation ability. This research model showed high prediction efficiency for VTE (AUC = 0.960). In this study, a validation data set consisting of 63 cases of VTE patients and 85 cases of non-VTE patients was selected for model validation. The accuracy of VTE risk warning model for VTE prediction was 81.8%. The high AUC of this prediction model may be related to the modeling data of VTE group and control group matched 1: 1. Of course, the incidence of VTE was relatively low in the actual clinical process, which required continuous improvement and adjustment in a wider range of later use in order to reach clinical value maximization. The sensitivity of this model to VTE early warning was significantly higher than that of Caprini risk assessment scale. For VTEs in life-threatening situation, early identification will benefit the most. Therefore, the warning model’s high sensitivity was in line with expectations. Although the degree of sensitivity and specific it was often difficult to achieve perfect synchronization state of ideal, the early warning model of VTE specificity was 5% lower than Caprini risk assessment scale. It indicated that some of the non-VTE patients with risk factors were identified by some early warning indicators, leading to a certain amount of false positives.

### Clinical status of VTE early warning mechanism

In order to take timely and effective measures to prevent the occurrence or further progress of VTE, clinicians and nurses should be kept informed of VTE early warning, including high risk of occurrence and early identification. There were many methods for clinical VTE evaluation, each with a certain scope of application and the results are barely satisfactory. A retrospective single-center study on patients who underwent thoracic surgery showed the areas under the receiver operating characteristic (ROC) curve of Caprini was 0.74 (*P* < 0.0001), Rogers 0.52 (*P* = 0.62), Padua 0.69 (*P* < 0.0001), and Khorana 0.64 (*P* = 0.0017), respectively [29]. In another study, ROC indicated that the Caprini score showed a significant but moderate relationship to VTE (AUC = 0.64; *p* = 0.004) [30]. Other studies had reached similar conclusion [31–33]. Though, many embedded VTE warning software has been developed and integrated with electronic medical record system but such software was mostly based on Caprini Risk Assessment Scale, or Padua Assessment Scale, etc. [33–36]. Vyas et al. [37] adopted the analysis way of Ishikawa Fishbone Diagram, and found that main reason for the improper prevention of DVT were the lacks of unified standard specifications, the computerized input system for doctors’ orders and effective risk assessment methods [38]. Also, others improved the Caprini Risk Assessment Scale [31, 32], but a lot of useful information in the electronic medical record system was not really used. These evaluation scales had not passed domestic large-scale clinical certification, and the accuracy and sensitivity of VTE screening were not very high. Therefore, the embedded automatic assessment and early warning system designed based on these scales usually have some inherent deficiencies.

### Clinical significance of VTE early warning model

The prevention and treatment of VTE is a hot topic in the medical field, and it’s also a difficult point in clinical work. The VTE prediction model were established with purpose of making accurate assessment and diagnosis of VTE in the first time and avoiding adulterating human factors as much as possible. We know that there is a lot of VTE-related information in the electronic medical record system [27, 33–35, 39–41], and such information needs to be further explored and fully utilized in the clinical VTE warning. The VTE risk warning model made full use of Caprini risk assessment scale, which was widely used in clinical medicine and surgery, with the important clinical symptoms and signs of VTE patients and laboratory examination indicators, to carry out comprehensive and multi-dimensional warning and achieve higher prediction efficiency. The work intensity of Chinese medical staff is very high, and it’s a great challenge to monitor patients’ conditions consistently. We screened six independent warning indicators except the Caprini score, including pleural chest pain, shortness of breath after exercise, chest tightness and shortness of breath, unilateral lower extremity pain, smoking, fibrinogen degradation product. We set up standard terms and captured the records of standard terms in electronic medical record system in order to establish electronic active alarm system which can prompt doctors and nurses to take timely responses. It is of great clinical importance to develop embedded electronic VTE active alarm systems based on VTE risk warning model.

### The deficiency and prospect of this research

We could not avoid the sample selectivity bias caused by the retrospective study. During this study, prothrombin time, D-dimer, and leukocytes in blood biochemical indicators were statistically important in univariate analysis, but they failed to enter the model during multivariate analysis. In addition, several articles had shown that platelets, inflammatory indicators, and the ratio of certain cell counts were also important in VTE early warning. Therefore, many blood biochemical indicators in clinical had potential value in the prediction and warning of VTE, which needs to be proved by more high-quality studies. This study only explored newly discovered independent warning indicators of VTE, and the mechanism of each warning indicator needs to be further studied. In addition, due to the limitation of various factors in the single-center study, the all-dimensional and multi-dimensional VTE risk warning model based on series of clinical comprehensive indicators needs to be constantly improved, verified and promoted in more centers and larger samples.

## Conclusions

In this study, VTE risk warning model includes seven independent risk factors, namely pleural chest pain, shortness of breath after exercise, chest tightness and shortness of breath, unilateral lower extremity pain, smoking, fibrinogen degradation product, Caprini score. A high early warning effect has been verified on VTE in hospitalized patients and the VTE risk warning model has certain clinical application value.

## Supplementary information

**Additional file 1.**

## Data Availability

All data generated or analysed during this study are included in this published article (and its supplementary information files).

## Abbreviations

VTE: Venous thromboembolism
ROC: Receiver operating characteristic
AUC: Area under curve
DVT: Deep venous thrombosis
PE: Pulmonary embolism
AHA: American heart association
PTS: Post-thrombotic syndrome
AT: Anticoagulant proteins
BMI: Body mass index
TP: True positive
FP: False positive
FN: False negative
TN: True negative
Acc: Accuracy
Sen: Sensitivity
PR: True positive rate
FNR: False negative rate
Spe: Specificity
TNR: True negative rate
FPR: False positive rate
LR: Likelihood ratio
